# Vital role of autophagy flux inhibition of placental trophoblast cells in pregnancy disorders induced by HEV infection

**DOI:** 10.1080/22221751.2023.2276336

**Published:** 2023-11-08

**Authors:** Yifei Yang, Bo Liu, Jijing Tian, Xuepeng Teng, Tianlong Liu

**Affiliations:** a National Key Laboratory of Veterinary Public Health and Safety, College of Veterinary Medicine, China Agricultural University, Beijing, People’s Republic of China; b Shandong Cancer Hospital and Institute, Shandong First Medical University and Shandong Academy of Medical Sciences, Jinan, People’s Republic of China

**Keywords:** Hepatitis E virus, autophagy flux, JEG-3, placenta, abortion

## Abstract

Hepatitis E virus (HEV) has become one of the important pathogens that threaten the global public health. Type 3 and 4 HEV are zoonotic, which can spread vertically and cause placental damage. At the same time, autophagy plays an important role in the process of embryo development and pregnancy maintenance. However, the relationship between HEV and autophagy, especially in the placenta tissue, has not been clarified. We found lower litter rates in HEV-infected female mice, with significant intrauterine abortion of the embryo (24.19%). To explore the effects of HEV infection on placenta autophagy, chorionic cells (JEG-3) and mice placenta have been employed as research objects, while the expression of autophagy-related proteins (ATGs) has been detected in JEG-3 cells with different times of HEV inoculation. The results demonstrated that the expression of protein LC3 decreased and p62 accumulated, meanwhile ATGs such as ATG4B, ATG5, and ATG9A in JEG-3 cells have decreased significantly. In addition, the maturation of autophagosomes, which referred to the process of the combination of autophagosomes and lysosomes was prevented by HEV infection as well. All processes of autophagic flux, which include the initiation, development, and maturation three stages, were suppressed in JEG-3 cells after HEV infection. Similarly, the protein and gene expression of LC3 were significantly decreased in the placenta of pregnant mice with HEV infection. In summary, our results suggested that HEV inhibited autophagy in JEG-3 cells and placenta of pregnant mice, which might be the important pathogenic mechanisms of HEV infection leading to embryo abortion.

## Introduction

Acute or chronic hepatitis caused by Hepatitis E Virus (HEV) infection is a global public health. At present, about 2 billion people live in areas where HEV is endemic, and more than 20 million people are infected HEV each year, leading to 70,000 deaths [1]. HEV belongs to the *Hepeviridae* family [2], among which *Orthohepevirus* A HEV is an important pathogen causing human public health problems and is the focus of recent research. *Orthohepevirus* A species includes 8 different genotypes which 1 and 2 are mainly prevalent in developing countries [3]. Genotypes 3, 4, and 7 are zoonotic and widely prevalent in industrialized countries [4]. HEV is a single-stranded positive-sense RNA virus with a gene sequence length of 6.4–7.2 kb. The genome contains 3 or 4 open reading frames (ORFs) [5]. HEV contains two forms which are the virion without capsule (neHEV) and wrapped with lipid outer membrane (eHEV) [6]. According to previous reports, the virion is eHEV when HEV is in the blood while the virion is neHEV when they are in faeces and bile [7,8].

Hepatitis E (HE) caused by HEV infection in fully immunized individuals is self-limited. However, it is serious to some patients which are immunocompromised, aged, or pregnant, and the mortality rate can be increased by causing outbreak hepatitis [9,10]. Pregnant women, a special susceptible group, have a significantly higher mortality rate after HEV infection, which has attracted wide attention worldwide. In 2001, an epidemiological survey of 469 pregnant women in the United Arab Emirates showed that the seroprevalence rate of HEV was 20%, and 30% of them had symptoms related to persistent infection. At the same time, pregnant women with high detected virus load had a significant tendency to vertical transmission [11]. In 2012, Germany also reported the first case of acute HEV-3 during pregnancy [12]. Recently, a survey of 990 pregnant women in Qingdao and Weihai, two cities in China, showed that the seroprevalence rate of HEV was 2.6% during the past ten years [13]. In view of the high prevalence of HEV and the special harm in pregnant women, chorionic trophoblast cells (JEG-3) and mouse placenta were selected as research subjects to explore the interaction mechanism between HEV and body.

Autophagy is an essential clearance mechanism for the body, which exists widely in eukaryotic cells, including physiological functions such as providing energy, maintaining cell homeostasis, and promoting apoptosis of abnormal cells [14,15]. Autophagy flux includes three stages containing the initiation of autophagy, the formation of autophagosome, and the degradation of autolysosome [16]. The whole process of autophagy flux is completed by ATGs coordination and intracellular components participation [17,18]. On the other hand, autophagy regulates the pregnancy process from three aspects of embryo, mother, and maternal immunity to maintain the balance of pregnancy [19]. Current studies have shown that the occurrence of pregnancy complications such as preterm delivery, eclampsia, and fetal development retardation is associated with abnormal autophagy [20,21]. Meanwhile, autophagy plays an important role in the proliferation, differentiation, and invasion of trophoblast cells as well as the reconstruction of vascular [22–24]. LC3, a marker protein of autophagy, and Beclin1 can be detected to be distributed widely in the placenta of pregnant women during the first trimester of pregnancy [25]. Abnormal autophagy in trophoblast cells will cause adverse pregnancy. Therefore, the regulation of autophagy is expected to become a breakthrough in the treatment of abnormal pregnancy [19]. In summary, we have chosen autophagy, a key point in abnormal pregnancy, to explore the interaction mechanism between HEV infection and JEG-3 cells in this study.

⁠Here, western blotting (WB) and real-time quantitative PCR (qRT-PCR) were applied to detect the expression of ATGs while transmission electron microscopy (TEM) and fluorescence microscopy were used to observe the formation of autophagosomes and autolysosomes in JEG-3 cells after HEV infection. Our results demonstrated that the protein and gene expression of LC3 decreased significantly together with the inhibition of ATGs such as ATG4B, ATG5, and ATG9A. In addition, the number of autophagosomes decreased and the degradation of autolysosomes was suppressed. In conclusion, three stages of autophagy flux which contain the autophagy activation, autophagosome formation, and autophagosome maturation were significantly inhibited in JEG-3 cells after HEV infection, indicating the normal physiological function of the cells was disrupted.

## Materials and methods

### Virus, cells, and animals

The HEV strain used in this study was GDC9, GenBank number FJ906895.1. JEG-3 was purchased from ATCC (Manassas, Virginia, USA). The ICR mice used in the experiments were purchased from Beijing Vital River Laboratory Animal Technology Co., Ltd. (Beijing, China) and were 8-weeks-old pregnant mice.

### Materials

Kits for HEV RNA extraction and PCR amplification were purchased from QIAGEN (Dusseldorf, Germany) and TaKaRa (Kusatsu, Shiga, Japan) respectively. TaqMan^TM^ Gene Expression Master Mix and *Powe*r^®^ SYBR Green PCR Master Mix were supplied by Applied Biosystems (Carlsbad, California, USA). Mouse anti-HEV monoclonal antibody was obtained from Merck (Darmstadt, Germany). Rabbit monoclonal to LC3B, ATG5, ATG16L1, ATG4B, ATG9A, and Beclin1 antibodies were contained in the autophagy marker antibody sampler panel which was purchased from Abcam (Cambridge, UK). Rabbit monoclonal to Bcl-2, goat Anti-Rabbit IgG H&L (HRP), goat anti-mouse IgG Fc (HRP), goat Anti-Mouse IgG H&L (Alexa Fluor^®^ 647) and were purchased from Abcam as well. SQSTM1/p62 mouse mAb was provided by Cell Signaling Technology (Boston, USA). Kit for autophagosomes detection was purchased from Abcam. Rapamycin (RAPA) and chloroquine (CQ) for autophagy induction and inhibition were supplied by MCE (New Jersey, USA), which have been stored and diluted according to the manufacturer’s recommendations. Ad-mCherry-GFP-LC3B, adenovirus employed as vector and LC3B labelled with two fluorescence signals, was obtained from Beyotime Biotechnology (Shanghai, China) and DAPI was provided by Solarbio (Beijing, China). The minimum essential medium (MEM) and fetal bovine serum (FBS) applied for cell culture were purchased from HyClone (Logan, Utah, USA). Trypsin and cell culture dishes were supplied by Gibco (Grand Island, NY, USA) and Corning (NY, USA), respectively.

### HEV isolation and load determination

The HEV strain was isolated from rabbit faeces, which was provided by the National Institutes for Food and Drug Control (Beijing, China) and purified over multiple generations. Rabbit faecal samples were resuspended with phosphate buffer saline (PBS) to prepare as a 20% (W/V) viral suspension and then centrifuged at 12,000 rpm, 4°C for 10 min. The viral suspension was filtered by syringe filters (0.22 μm, Millipore, Massachusetts, USA) and then stored at −80°C. Plasmid standard curves were constructed and the viral load of HEV in suspension was detected by real-time quantitative PCR with kits previously described in Materials. The HEV titre in suspension was 5.0 × 10^6^ copies/mL. The plasmid and primer sequences for qRT-PCR are presented in Supplementary Table 1.

### JEG-3 cells and mouse placentas with HEV infection

A total of 2 × 10^6^ JEG-3 cells were inoculated with 1 × 10^5^ copies HEV and the adherent cells were harvested after 24 h of co-incubation. Pregnant ICR mice with similar body weight were injected with HEV through tail vein, which each mouse was challenged with 1 × 10^6^ copies HEV. The mice were sacrificed at 3, 6, 12, and 24 h after challenge and the uterine and placental tissues were isolated. The tissues were partially fixed in 4% paraformaldehyde and partially frozen in a refrigerator at –80°C for subsequent experiments. Pregnancy identification of ICR mice is presented in Supplementary Figure 1 (Fig. S1).

### PCR

Trizol, which was provided by Invitrogen (Carlsbad, California, USA), was applied to extract RNA from JEG-3 cells and mouse placenta tissues with HEV infection. RNA was reverse-transcribed into cDNA and amplified with the kit mentioned in Materials, the instrument for PCR purchased from BIO RAD (California, USA). The PCR products were subjected to nucleic acid gel electrophoresis and photographed by gel image system Tanon 1600 (Shanghai, China). The primer sequences of HEV for virus identification by RT-PCR can be seen in Supplementary Table 2.

To determine the changes of LC3 mRNA in JEG-3 cells and mouse placenta tissues with HEV treatment, relative fluorescence quantitative PCR was performed by using *Power*^®^ SYBR Green PCR Master Mix, which was already mentioned in Materials. The instrument applied for qRT-PCR was purchased from Applied Biosystems (Carlsbad, California, USA). The primer sequences of LC3 and internal reference are presented in Supplementary Table 2 as well.

### Immunofluorescence staining (IFA)

JEG-3 cells were maintained in MEM supplemented with 10% FBS in the cell incubator with 5% CO_2_ at 37°C. Cells were inoculated with 1 × 10^5^ copies HEV when they had grown to 60–70% in 35 mm glassy bottom dishes, continued to co-culture with HEV for 24 h. The medium was discarded and the cells were washed with cold PBS for 3 times. 4% paraformaldehyde was added to cells and fixed for 15 min. After washing, serum was added to the cells and incubated for 30 min to block antigens. The cells were incubated with a mouse anti-HEV monoclonal antibody (Millipore, MAB8002, 1:200) at 4°C overnight. The next day, after washing with PBS, the cells were incubated with goat anti-mouse IgG H&L (Alexa Fluor^®^ 647) for 45 min at room temperature. After the supernatant discarded and the dishes naturally air-dried, antifade mounting medium which was provided by Beyotime Biotechnology (Shanghai, China) was added to the cells. Fluorescence microscope, the instrument provided by Leica (Heidelberg, Germany) was operated to observe and take photos. The cell culture medium, serum, antibodies, and other materials used in this part have been mentioned before.

### Immunocytochemistry (IHC)

Mouse placenta tissues were fixed in 4% paraformaldehyde for more than 3 days and paraffin sections were prepared with a thickness of 3 μm. The paraffin sections were dewaxed to water, according to the basic haematoxylin and eosin (H&E) staining protocol, and then were placed in buffer which completely immersed the sections for antigen repair. Antigen repair was performed by microwave heating, high heat for 5 min, low heat for 20 min, and then natural cooling to room temperature. Endogenous peroxidase reagent was added to the sections and incubated for 10 min at room temperature. After washing with PBS, the sections were incubated with the anti-HEV (Millipore, MAB8002, 1:200) and anti-LC3 antibody diluent (abcam, ab192890, 1:200) at 4°C overnight. The next day, an appropriate amount of goat anti-mouse or rabbit IgG (HRP) reagent was incubated with the sections for 20 min at room temperature. After washing, the sections were incubated with the chromogenic agent (DAB) and observed under a microscope. The sections were placed in PBS to terminate the colour development. The nuclei were stained with haematoxylin and then the sections were dehydrated according to the staining protocol. Finally, the sections were observed and photographed under a microscope, which was purchased from Nikon (Chiyoda, Tokyo, Japan).

### Western blot

JEG-3 cells and mouse placenta tissues with HEV infection were treated with RIPA lysate (Solarbio, Beijing, China) and lysed for 20 min on ice. BCA protein quantification kit, which was provided by Thermo Fisher Scientific (Waltham, USA), was applied to measure and adjust the protein concentration of the samples in each group. The sample was heated with boiling water for 10 min after loading buffer (Cell Signaling Technology, Boston, USA) was added. Protein samples were separated by SDS‒PAGE (10% Bis-Tris Gel) and transferred to polyvinylidene fluoride (PVDF) membranes, which were obtained from Millipore (Bedford, USA). The membrane was transferred at a constant current of 200 mA for 50‒100 min, depending on the size of the target protein. After washing, the PVDF membranes were immersed in PBST containing 5% skim milk powder (Mengniu, Inner Mongolia, China) for 60 min at room temperature and then incubated with primary antibodies at a dilution of 1:1000 at 4°C overnight. The next day, after washing with PBST for three times, the PVDF membranes were incubated with goat anti-rabbit or mouse IgG (HRP) for 50 min at room temperature. Washing with PBST again, the PVDF membranes were developed with immobilon classico western HRP substrate (Millipore, Bedford, USA) and photographed by Tanon 5200 (Shanghai, China).

### Statistical analysis

All charts in this experiment were performed by GraphPad Prism 8 software (GraphPad Software, Inc., CA, USA), and SPSS Statistics 25 (IBM, Inc., NY, USA) was applied for statistical analysis. All experimental data were presented as the mean ± SD. Student’s *t*-test and a one-way ANOVA were used to compare multiple experimental groups. * *P* < .05 was considered to be statistically significant.

## Results

### HEV genotype 3 infection caused fetal loss in pregnant ICR mice

**Figure 1.**
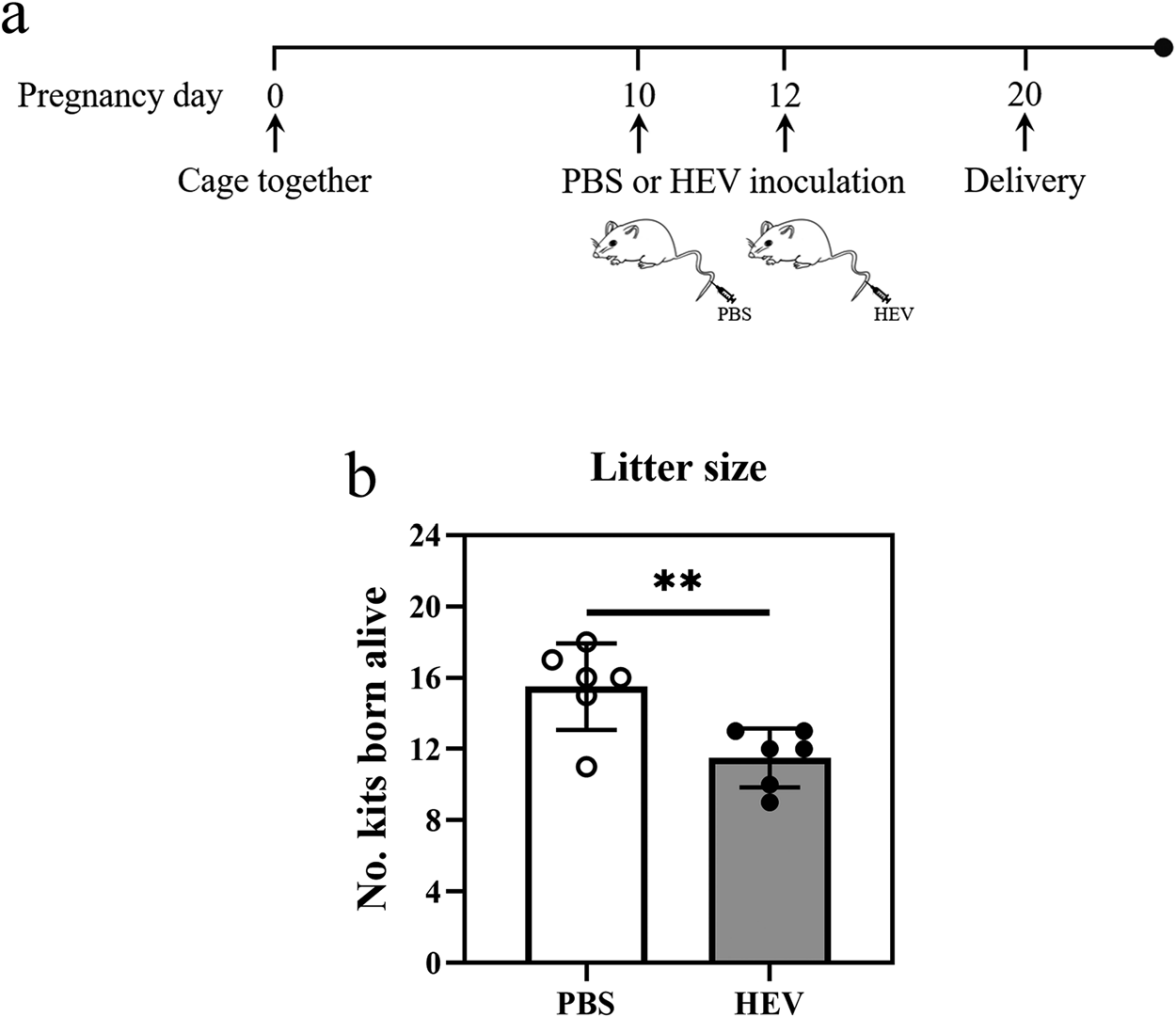
HEV genotype 3 infection caused fetal loss in pregnant ICR mice. (a) Schematic of the experimental design for HEV-3ra infection in pregnant ICR mice. Specific-pathogen-free (SPF) pregnant ICR mice were inoculated PBS (control group, *n* = 6) or 1 × 10^6^ copies HEV (infection group, *n* = 6) via tail vein injection on the 10th and 12th day of mice gestation respectively. (b) Litter sizes in pregnant ICR mice with PBS or HEV inoculation. Litter size in pregnant ICR mice significantly decreased after HEV exposure, with a fetal loss of 24.19% compared to the control group. Statistical significance was determined by two-tailed unpaired Student’s *t*-test (**, *P *< .01). Bars indicate mean ± SEM (*n* = 6).

Adverse pregnancy outcomes have been reported in 38.5% of HEV-infected pregnant women, which causes significant placental damage and vertical transmission [26]. Similarly, embryo abortion has occurred in HEV-infected pregnant rabbits and BALB/c mice as well [27,28]. ICR mice, a common laboratory animal model, were attempted to be applied in this experiment to construct a model of abortion induced by HEV infection. Since mouse was not a susceptible animal model for HEV infection, we conducted two challenges with 1 × 10^6^ copies HEV each time via tail vein injection on the 10th and 12th day of mice gestation, respectively (Figure 1(a)). The number of newborns between the groups of HEV or PBS injections was compared after delivery. Our results exhibited that the litter sizes of HEV-inoculated mice had significantly lower than that of controls (Figure 1(b), Table S3), and the rate of embryonic abortion was 24.19%, similar to the results of previous studies [28]. It was also worth noting that one mother mouse in the HEV infection group experienced premature birth with a litter size of 9. In addition, the weight of newborn mice in the experimental groups were significantly lower than that of the control groups during two mouths record (Fig. S7).

### HEV genotype 3 infected JEG-3 cells and placenta of pregnant ICR mice

**Figure 2.**
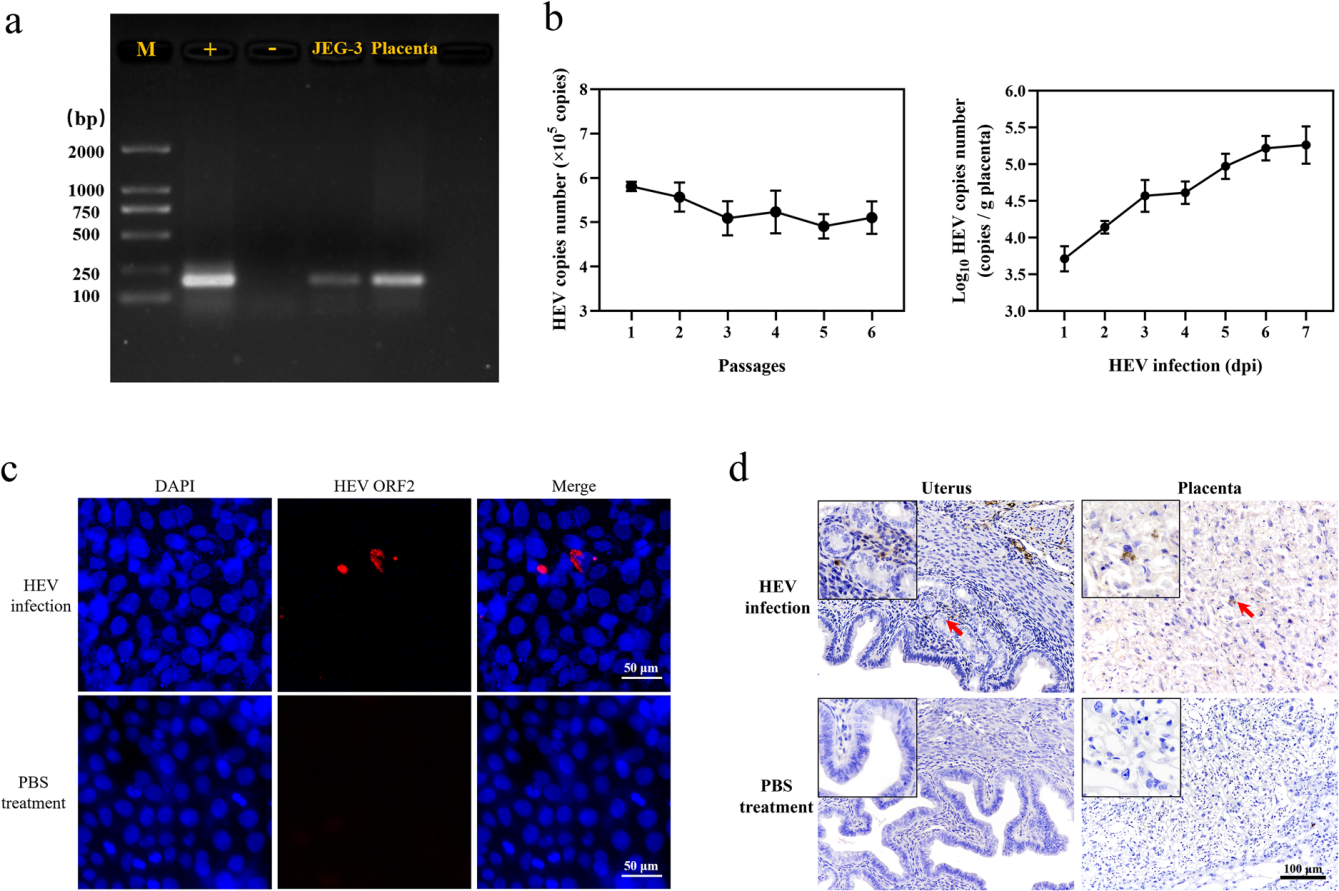
HEV genotype 3 infected JEG-3 cells and placenta of pregnant ICR mice. (a) Nucleic acid gel electrophoresis of HEV in JEG-3 cells and placenta. HEV Kernow-C1/p6 strain and PBS were applied as positive control (+) and negative control (−) respectively. (b) Detection of HEV RNA in JEG-3 cells and placenta by qRT-PCR. Bars indicate mean ± SEM, *n* = 3. (c) Immunoﬂuorescence detection of HEV ORF2 antigen in JEG-3 cells. Nuclei stained by DAPI. Scale bar represents 50 μm. (d) Immunohistochemical staining of HEV ORF2 in mouse uterus and placental cells. Scale bars represent 50 μm (c) and 100 μm (d).

In view of the adverse pregnancy and relatively high mortality of pregnant women caused by HEV, human trophoblast cells (JEG-3) were selected as an in vitro model. In addition, it has been reported that HEV can infect pregnant mice, causing miscarriage and uterine injury in female mice [29]. Therefore, whether HEV-3 could infect JEG-3 cells and the placenta of pregnant mice was first investigated. The HEV suspension was inoculated in JEG-3 cells (1 × 10^5^ copies) and ICR mice (1 × 10^6^ copies) by tail vein injection separately. HEV positivity was detected in both serum and liver of the mice, and small foci of inflammation dominated by lymphocytes and plasma cells were observed in haematoxylin eosin-stained sections of the liver, indicating that pregnant ICR mice could be infected with HEV (Fig. S2). Cells and placenta tissue were collected after 24 h post HEV inoculation (hpi). Nucleic acid gel electrophoresis showed a positive band at 188 bp (Figure 2(a)). Meanwhile, after HEV infection, HEV RNA could be detected in JEG-3 cells which were serially passaged for at least 6 passages and the placenta of mice within 7 days post inoculation (dpi) (Figure 2(b)). The results of IFA and IHC revealed that HEV was active and had the ability to enter cells, uterus, and placenta, mainly distributed in the cytoplasm of JEG-3 cells, the epithelial cells surrounding the uterine glands and the trophoblast cells of the placenta (Figure 2(c, d)).

### HEV genotype 3 infection inhibited autophagy in JEG-3 cells

**Figure 3.**
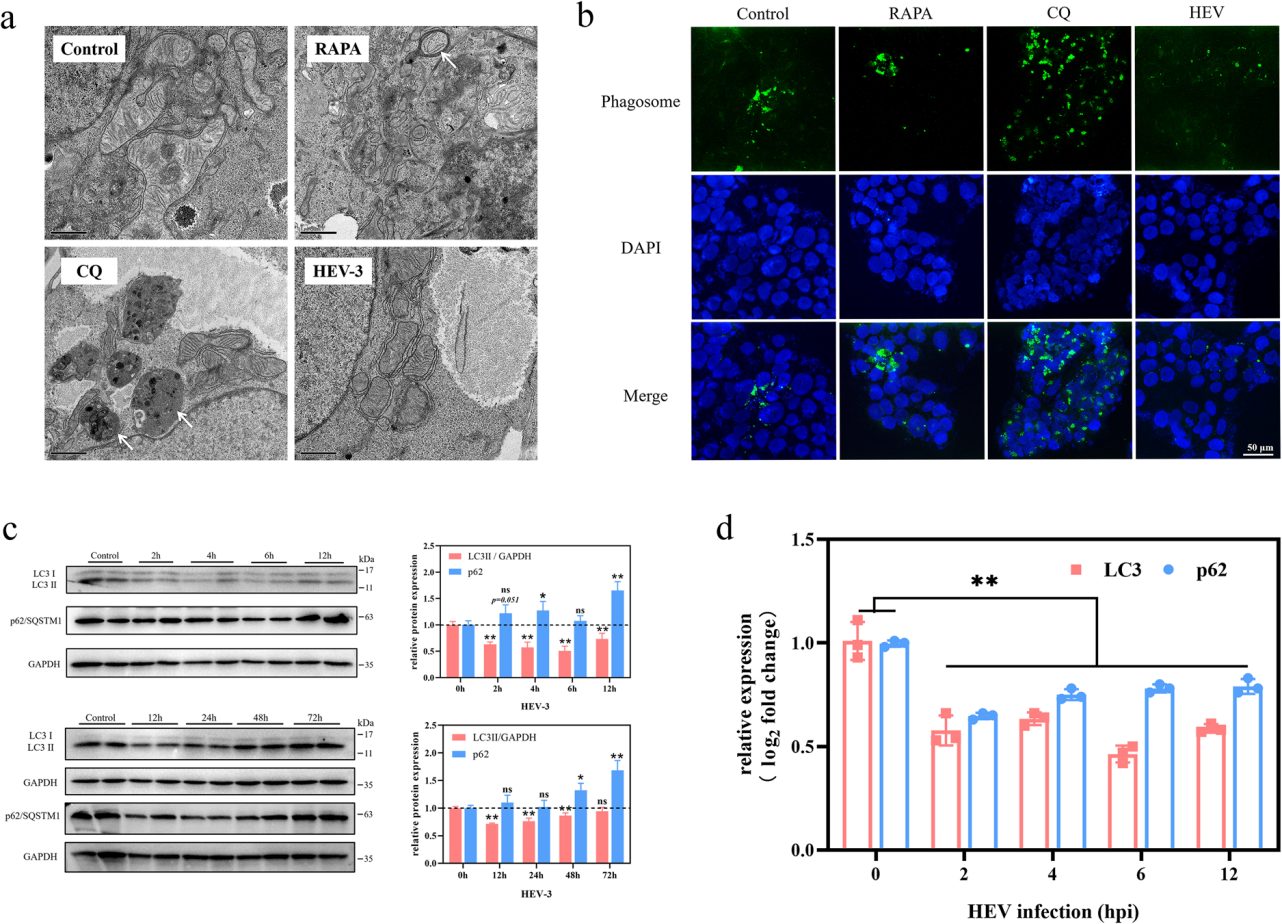
Autophagy inhibition in JEG-3 cells with HEV genotype 3 infection. (a) TEM of phagosome/autophagosome (arrow) and (b) fluorescent staining of autophagic vacuoles in JEG-3 cells treated with PBS, RAPA (100 nM), CQ (50 μM), and HEV. Nuclei stained by DAPI. Scale bars represent 1 or 50 μm. (c) Western blot and (d) qRT-PCR of LC3 and p62 in JEG-3 cells with different time HEV infection. Bars indicate mean ± SEM, *n* = 3. *P < .05, **P < .01.

Double-membrane-structured autophagosomes, which can be observed under transmission electron microscopy (TEM), are considered markers of intracellular autophagy [15]. Therefore, we first observed changes in the number of autophagosomes in JEG-3 cells after HEV infection. Double-membrane autophagosomes (arrows) were observed in JEG-3 cells treated with rapamycin (RAPA, 100 nM, 24 hpi). In contrast, autophagosomes were hardly observed in cells from the control and HEV-infected groups. Chloroquine (CQ) is often applied as an autophagy inhibitor, inhibiting the maturation and degradation of autolysosome [30]. Under the TEM, a large number of monolayer-structured phagosomes (arrows) could be seen in JEG-3 cells treated with CQ (50 μM, 24 hpi) (Figure 3(a)). To better evaluate the changes in the number of autophagic vacuoles in JEG-3 cells after HEV infection, monodansylcadaverine (MDC), a conventional fluorescent probe, was applied as a fluorescent marker for autophagosomes staining. Fluorescence signals were significantly enhanced in both RAPA and CQ-treated cells, whereas autophagic flux was inhibited in HEV-infected cells (Figure 3(b), S3). Cells with acute HEV infection (0−12 hpi) and persistent infection (12−72 hpi) were collected, respectively and then the changes in expression of LC3 and p62 protein, which were markers of autophagy, were detected by western blotting (WB). The LC3 protein is involved in the formation and extension of the autophagosome membrane, and the dynamic process of esterification of LC3-I to LC3-II usually indicates intracellular autophagy [31]. Hence, the ratio of LC3-II to an internal reference is applied to measure changes in intracellular autophagy. Besides, p62 protein is mainly involved in the fusion process of autophagosome and lysosome, in which the amount of p62 protein is reduced due to depletion. Our results demonstrated that after HEV infection, LC3-II/GAPDH was significantly lower (2−72 hpi) and p62 protein was significantly increased (48−72 hpi) compared to the control group, which indicated that autophagy in JEG-3 cells was inhibited (Figure 3(c), S6, **P < .01, *P < .05). We further examined the changes in the mRNA levels of these two proteins. The results showed that both proteins were significantly reduced at the genomic transcription level compared with the control group, which suggested that HEV down-regulated the transcription of autophagy-related genes to inhibit autophagy in JEG-3 cells (Figure 3(d)).

### HEV genotype 3 infection inhibited autophagy flux in JEG-3 cells

**Figure 4.**
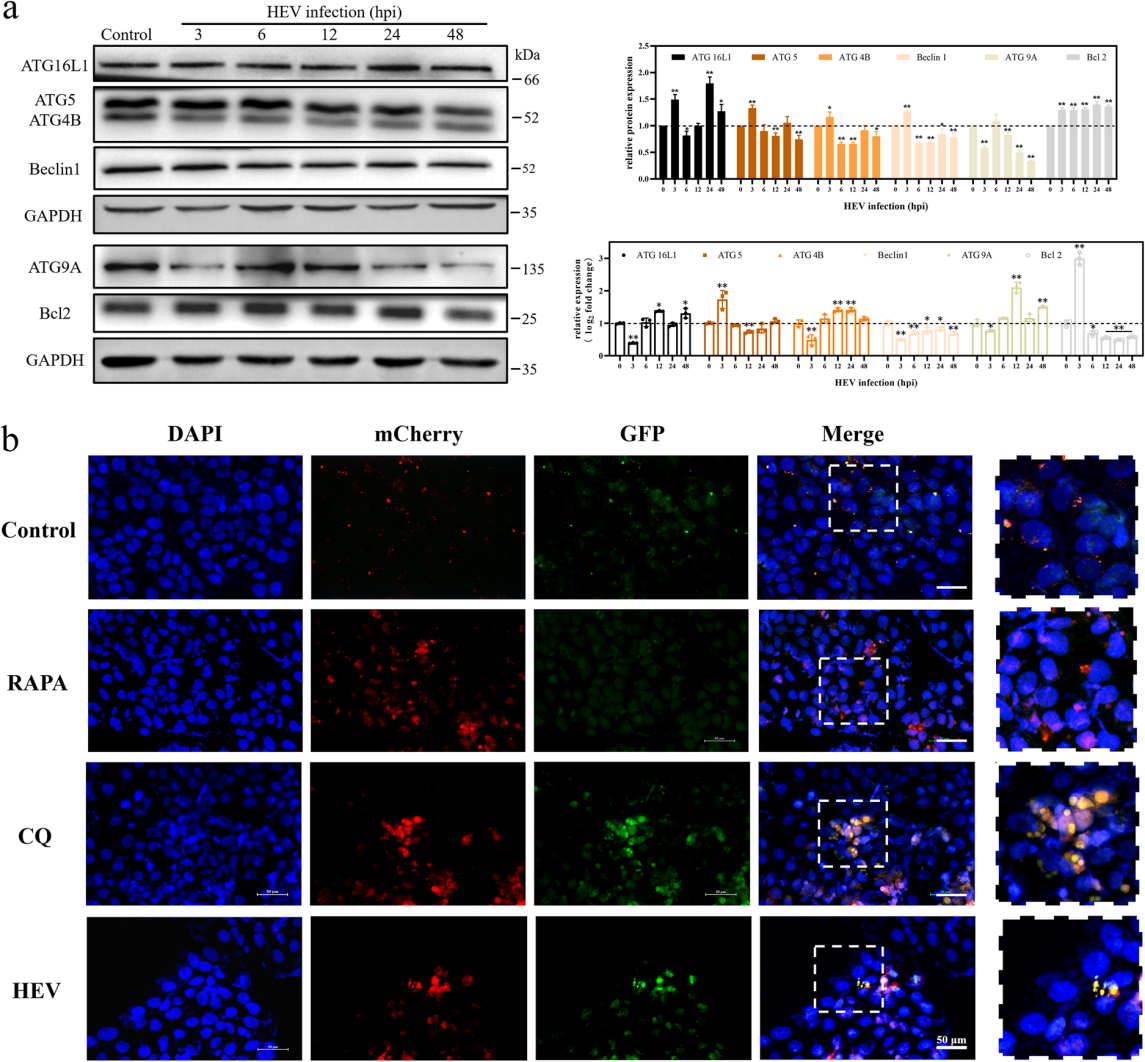
Autophagy flux inhibition in JEG-3 cells with HEV genotype 3 infection. (a) Western blot of ATG16L1, ATG5, ATG4B, Beclin1, ATG9A, and Bcl2 protein in JEG-3 cells with different time HEV infection. Bars indicate mean ± SEM, *n* = 3. *P < .05, **P < .01 (b) Fluorescence photos of JEG-3 cells transfected with mCherry-GFP-LC3 plasmid treated with PBS, RAPA (100 nM), CQ (50 μM) and HEV. Nuclei stained by DAPI. Scale bar represents 50 μm.

Autophagy flux summarizes the three stages of autophagy occurrence, development, and maturation, in which autophagy-related proteins (ATGs) regulate each stage [32,33]. Among them, Beclin1 and Bcl2 proteins, which antagonize each other to regulate autophagy [34], are involved in the initiation of autophagy. ATG4B, ATG5, and ATG16L1 proteins are relevant to the expansion and formation of autophagosome membranes. WB and qRT-PCR were applied to detect the expression of ATGs within 48 hpi of HEV infection. Beclin1 protein expression decreased and Bcl2 increased, indicating that the autophagy initiation process was inhibited. The gene expression of Beclin1 and Bcl2 was also consistent. Bcl2, in particular, exhibited a highly significant elevation in the first 3 hpi of HEV. At the same time, both the protein and gene expression of ATG5 and ATG4B displayed a downward trend, while the ATG16L1 protein expression increased. It is worth noting that the increase of ATG16L1 protein expression cannot indicate that autophagy is enhanced. Because the expression level of ATG16L1 protein is not always related to autophagy, various factors affecting its protein expression [35]. In addition, ATG9A protein, the only known transmembrane ATG protein, is an essential protein that regulates autophagy in eukaryotes and is closely related to the occurrence and development of autophagy [36]. The results revealed that the expression of ATG9A protein decreased significantly within 12−48 hpi of HEV infection (Figure 4(a), **P < .01, *P < .05). The above data indicated that the initiation and development of autophagy in JEG-3 cells were inhibited by HEV infection.

To further explore the effect of HEV infection on the autolysosome maturation process, Ad-mCherry-GFP-LC3B, a dual fluorescent reporter system, was transfected into JEG-3 cells. Among this, the red fluorescence of mCherry was relatively stable in an acidic environment, while the green fluorescence of GFP was easily quenched by acid. When the autolysosome matured, the pH value in the autolysosome decreased and became acidic due to the cleavage of the lysosome. Our results demonstrated that the fluorescence images of HEV infection for 24 h were similar to that after CQ treatment. Both red and green fluorescence were clearly visible, with yellow fluorescence after merging (Figure 4(b)).

### HEV genotype 3 infection inhibited autophagy in the placenta of pregnant ICR mice

**Figure 5.**
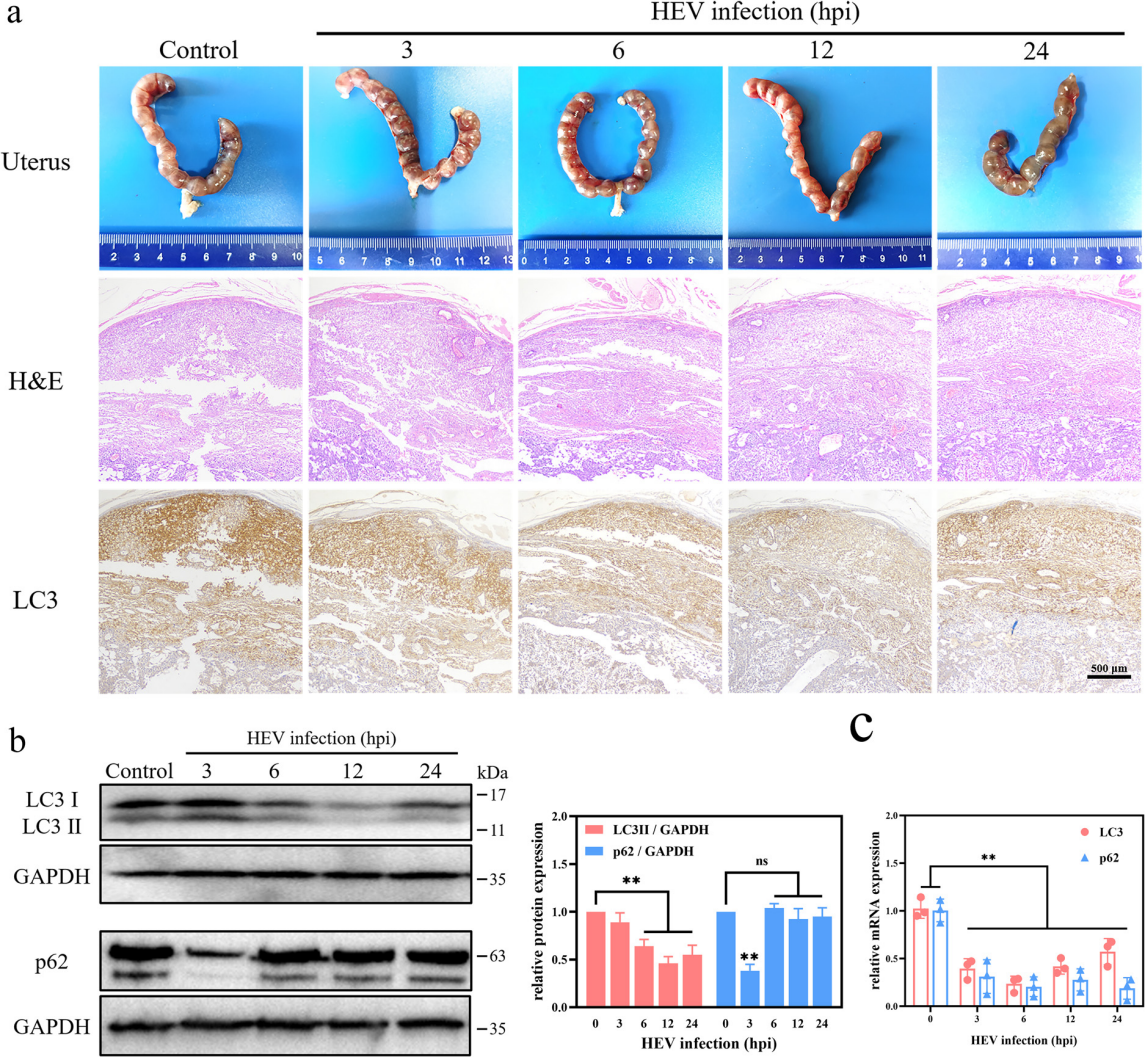
Autophagy inhibition in the placenta of pregnant ICR mice with HEV genotype 3 infection. (a) Images of uterus, H&E staining, LC3 immunohistochemical staining. (b) Western blot and (c) qRT-PCR of LC3 and p62 in the placenta with different time HEV infection. Bars indicate mean ± SEM, *n* = 3. **P < .01.

In order to find out the effect of HEV on autophagy of placental tissue, 10-day gestational ICR mice were injected with 1 × 10^6^ copies of HEV by tail vein injection. In view of the fact that autophagy often occurs in the early stage of cell stimulation, which is an adaptive response of cells to changes in the external environment [37]. Therefore, mice were sacrificed at 3, 6, 12, and 24 h after HEV challenge and the placental tissue was collected. There was no obvious placental tissue damage in mice within 24 h of HEV inoculation, but the expression of LC3 protein in placenta tissue decreased. There was a significant difference between the 12 hpi of HEV infection group and the control group (Figure 5(a), S4, **P < .01, *P < .05). Similarly, WB results also revealed that the LC3 protein significantly decreased after HEV infection within 6−24 h (Figure 5(b), **P < .01). The amount of LC3 mRNA in placental tissue was also significantly down-regulated (Figure 5(c), **P < .01). This was consistent with the experimental results in JEG-3 cells, indicating that HEV inhibited autophagy in mouse placental by down-regulating the LC3 gene transcription. The protein and gene expression of p62 in mouse placenta were also examined. However, a significant decrease of p62 protein expression could be found after 3 hpi of HEV, which was not consistent with that in JEG-3 cells. It was speculated that autophagy in mouse placenta was likely promoted in the early stages and then quickly inhibited, especially under the condition where gene expression was significantly suppressed. And the p62 exhibited significant inhibition at the gene level but not at the protein level after HEV infection for 6−24 h, which could be attributed to the blockage of p62 protein degradation. This is also a proof of p62 protein accumulation and autophagy inhibition.

## Discussion

HE is self-limiting, and most people do not have clinical symptoms or symptoms resolve spontaneously within a very short period of time after HEV infection [38]. Whereas, infection with HEV often results in more serious consequences for immuno-compromised or some special groups. Pregnant women, especially those in the second and third trimesters, have a very high chance of developing fulminant hepatitis, and the mortality rate increases to 30%. At the same time, HEV can be transmitted vertically and lead to adverse pregnancy such as premature birth, miscarriage, fetal dysplasia, etc. [39]. Similarly, HEV can also spread vertically in animals such as rabbits and mice, and damage their reproductive system, causing uterine injury and abortion [27,29,40,41]. However, the mechanism of how the virus damages the reproductive system and causes adverse pregnancy remains unknown.

Autophagy is a conservative and important defensive response of cells to external stimuli, which is used to remove damaged organelles and metabolic wastes to maintain cellular homeostasis [42]. Simultaneously, autophagy also plays an irreplaceable role in the development of body and cell differentiation, especially in the process of embryonic maturation and trophoblast cell differentiation [19]. Abnormal autophagy may lead to placenta-related diseases [43,44]. Therefore, we attempted to explore the effect of HEV infection on placenta from the perspective of autophagy. It has been reported that a decline in mice litter size often represents intrauterine fetal death or absorption, which can be considered a miscarriage [28,45]. In our study, pregnant ICR mice were infected with HEV by tail vein injection, leading to a significant reduction in the number of newborns and an abortion rate of 24.19% (Figure 1(b)). It has been hypothesized that autophagy might be one of the potential mechanisms of embryonic abortion caused by HEV infection. Hence, we first detected the expression of LC3, a marker protein of autophagy, with HEV infection in vitro and in vivo. The experimental results preliminarily demonstrated that the autophagy in cells or placental tissue was significantly decreased, whether it was acute or persistent infection. Afterwards, since autophagy is a continuous process, also known as autophagic flux, we wanted to further understand which process of autophagy was inhibited by HEV. The regulatory proteins in autophagic flux have been examined and the results illustrated that HEV inhibited all processes in autophagy flux. First of all, Beclin1 and Bcl2 interact to regulate the initiation of autophagy [34]. Beclin1 is binding with Bcl-2 when autophagy is inhibited, and when those two proteins separate with each other, autophagy initiates. After JEG-3 cells inoculated with HEV, the protein expression of Beclin1 decreased while Bcl-2 increased. Secondly, LC3 is an important raw material for the formation of autophagosome membranes, while ATG4B acts as a catalyst for the esterification process of LC3 [46,47]. The expression levels of both were significantly reduced after HEV infection, revealing that the development of autophagy was also inhibited. Finally, according to the abnormal accumulation of p62, of which mRNA transcription was down-regulated while its protein content was increased, and the tracking of the terminal stage of autophagy by the dual fluorescent reporter system mCherry-GFP-LC3, it was concluded that the maturation and degradation of autolysosome was inhibited by HEV. In general, our results clarified that the whole process of autophagy flux in JEG-3 cells was inhibited by HEV infection. This is not consistent with previous studies, which is speculated that there are differences in the choice of HEV genotypes and cell lines [48,49]. Decreased autophagy may be more damaging when it occurs in trophoblast cells, whose invasive function is dependent on intracellular levels of autophagy, which is strongly associated with miscarriage [19]. Nevertheless, the scientific data underpinning this section still needs to be further expanded.

Notably, two interesting phenomena were discovered during our experiments. In the first place, the transcription of LC3 performed a significant downward trend in both JEG-3 cells and mouse placenta (Figure 3(d), Figure 5(c)). It was initially speculated that the inhibitory effect of HEV on autophagy was based on the binding or degradation of LC3, a key autophagy protein. However, the obvious down-regulation of its transcription level suggested that HEV might have an impact on its upstream signalling pathway, which deserves further study. Another interesting finding was that the expression of ATG9A protein was significantly decreased during HEV infection. ATG9A protein is the only known transmembrane autophagy regulator protein, and is an essential protein for regulating autophagy in eukaryotes [50,51]. ATG9A regulates the occurrence and development of autophagy mainly by circulating back and forth between preautophagosomal structure (PAS), the autophagosome assembly area, and the peripheral “reservoir” which contains components of autophagosome membrane formation [36]. In addition, the localization of ATG9A is regulated by intracellular sphingomyelin level. When overexpression of sphingomyelin occurs, ATG9A is trapped in the paranuclear circulation system and cannot be recruited to the autophagosome membrane, resulting in impaired autophagy membrane closure and inhibition of autophagy [52]. Our data demonstrated that the expression of ATG9A protein gradually decreased with time after HEV infection of JEG-3 cells. We speculated that HEV may disturb the balance of the cell’s biofilm system and inhibit autophagy. Sphingomyelin and cholesterol, as the main lipid molecules constituting the biological membrane, regulate and balance each other [53]. Therefore, HEV may inhibit intracellular autophagy by interfering cholesterol formation and overexpressing sphingomyelin, which further obstructs the formation of autophagosome membrane. A recent study on HEV therapy also mentioned that HEV infection leads to a decrease in intracellular cholesterol levels [54]. However, due to the difficulties and obstacles in the current detection technology of phospholipid molecules, only conjectures and discussions are made here and more data needed to be demonstrated.

In conclusion, HEV genotype 3 could replicate continuously in JEG-3 cells and mouse placental tissue, as well as inhibit the occurrence and development of autophagy in JEG-3 cells, resulting in decreased expression of ATG proteins such as LC3, Bclin1, ATG4B, ATG5, ATG9A, and accumulation of p62. Besides, HEV inhibited the maturation of autolysosomes. Inhibition of autophagy in trophoblast cells and placental caused by HEV infection may be one of the vital mechanisms leading to embryonic abortion.

## Supplementary materials

The following are available online at https://doi.org/10.1080/22221751.2023.2276336, Figure Table S1: The plasmid and primer sequences, Table S2: The primer sequences for PCR, Table S3: The number of kits born alive in PBS and HEV inoculated pregnant ICR mouse groups, Table S4. Detection of viral load of HEV in JEG-3 cells and placental tissue by qRT-PCR, Fig. S1: Pregnancy identification of ICR mice by ultrasound, Fig. S2: Detection of HEV in the liver and serum of pregnant ICR mice with HEV infection, Fig. S3: Quantitative analysis of GFP fluorescence signals in JEG-3 cells, Fig. S4: Quantitative analysis of LC3 IHC in mouse placenta, Fig. S5: Effective JEG-3 cells autophagy induction and inhibition with drugs treatment, Fig. S6: Inhibition of autophagy in JEG-3 cells with HEV inoculation, Fig. S7: Mother mice with HEV injection produced a significantly slower body weight gain in their litters.

## Animal ethical statement

Animal experiments were approved by the Animal Ethics Committee of China Agricultural University (approval number 202,203,014).

## Author contributions

Conceptualization, T Liu, X Teng, and Y Yang.; methodology, Y Yang, J Tian, and B Liu.; formal analysis, Y Yang and X Teng.; resources, J Tian. and T Liu.; writing, Y Yang. and B Liu. All authors have read, commented upon, and approved the final article.

## Supplementary Material

Supplementary_tables_and_figures
