# Supplementary material for: Vital role of autophagy flux inhibition of placental trophoblast cells in pregnancy disorders induced by HEV infection
Source: Emerg Microbes Infect. 2023 Nov 8;12(2):2276336. doi: 10.1080/22221751.2023.2276336 (PMC10796124; doi:10.1080/22221751.2023.2276336)
Supplement: Supplementary_tables_and_figures [file TEMI_A_2276336_SM3936.docx]

**Supplementary Table 1. The plasmid and primer sequences**

| Genes | Sequences (5’ to 3’) |
| --- | --- |
| The plasmid | cgaatgcatctagatccggtcagccgtctggccgtcgccgcgggcggcgcagcggcggtgccggcggtggtttctggggtgaccgggttgattctcagcccttcgccctcccctatattcatccaaccaaccccttcgcatctgacataccaaccgcagccgggtctggagctcgccctcggcagatcggatcccgggcc |
| HEV | F: GGTGGTTTCTGGGGTGAC  R: AGGGGTTGGTTGGATGAA  P: FAM- TGATTCTCAGCCCTTCGC-TAMRA |

**Supplementary Table 2. The primer sequences for PCR**

| Genes | Sequences (5’ to 3’) |
| --- | --- |
| HEV | P1: CTGTTTAAYCTTGCTGACAC  P2: WGARAGCCAAAGCACATC  P3: GACAGAATTGATTTCGYCG  P4: TGTTGGTTRTCATAATCCTG  P5: TGCTGGTTATCGTAATCCTG |
| LC3 | F: GAGAAGACCTTCAAGCAG  R: GAGGCATAGACCATGTACAG |
| p62 | F: ATTGCCGTCTCCGTCTCG  R: CGACGTTGTGGACGTTTACC |
| Actin | F: TGGCTCCTAGCACCATGAAGAT  R: GGTGGACAGTGAGGCCAGGAT |

**Supplementary Table 3. The number of kits born alive in PBS and HEV inoculated pregnant ICR mouse groups**

| Group | Female mouse ID | No. kits born alive | Average litter size |
| --- | --- | --- | --- |
| PBS | B1 | 16 | 15.5 |
|  | B2 | 18 |  |
|  | B3 | 11 |  |
|  | B4 | 17 |  |
|  | B5 | 15 |  |
|  | B6 | 16 |  |
| HEV | L1 | 12 | 11.5 |
|  | L2 | 10 |  |
|  | L3 | 13 |  |
|  | L4^a^ | 9 |  |
|  | L5 | 13 |  |
|  | L6 | 12 |  |

L4^a^, the female mouse numbered L4 showed premature labor, giving birth on the 18th day after mating, while all other females gave birth on the 20th day after mating.

**Supplementary Table 4. Detection of viral load of HEV in JEG-3 cells and placental tissue by qRT-PCR**

| Samples | Passage | Viral load data (log_10_ copies of HEV RNA/mL) | | |
| --- | --- | --- | --- | --- |
| JEG-3 cells | P1 | 5.59 | 5.58 | 5.57 |
|  | P2 | 5.59 | 5.56 | 5.52 |
|  | P3 | 5.55 | 5.51 | 5.47 |
|  | P4 | 5.58 | 5.51 | 5.49 |
|  | P5 | 5.52 | 5.49 | 5.47 |
|  | P6 | 5.55 | 5.50 | 5.48 |
| Samples | dpi^a^ of HEV | Viral load data (log_10_ copies of HEV RNA/g placenta) | | |
| Placenta | 1 | 3.63 | 3.91 | 3.60 |
|  | 2 | 4.16 | 4.22 | 4.05 |
|  | 3 | 4.45 | 4.44 | 4.82 |
|  | 4 | 4.73 | 4.67 | 4.44 |
|  | 5 | 5.13 | 4.79 | 5.00 |
|  | 6 | 5.30 | 5.33 | 5.03 |
|  | 7 | 5.47 | 4.98 | 5.34 |

dpi^a^, days post-inoculation.

**Supplementary Figure 1**


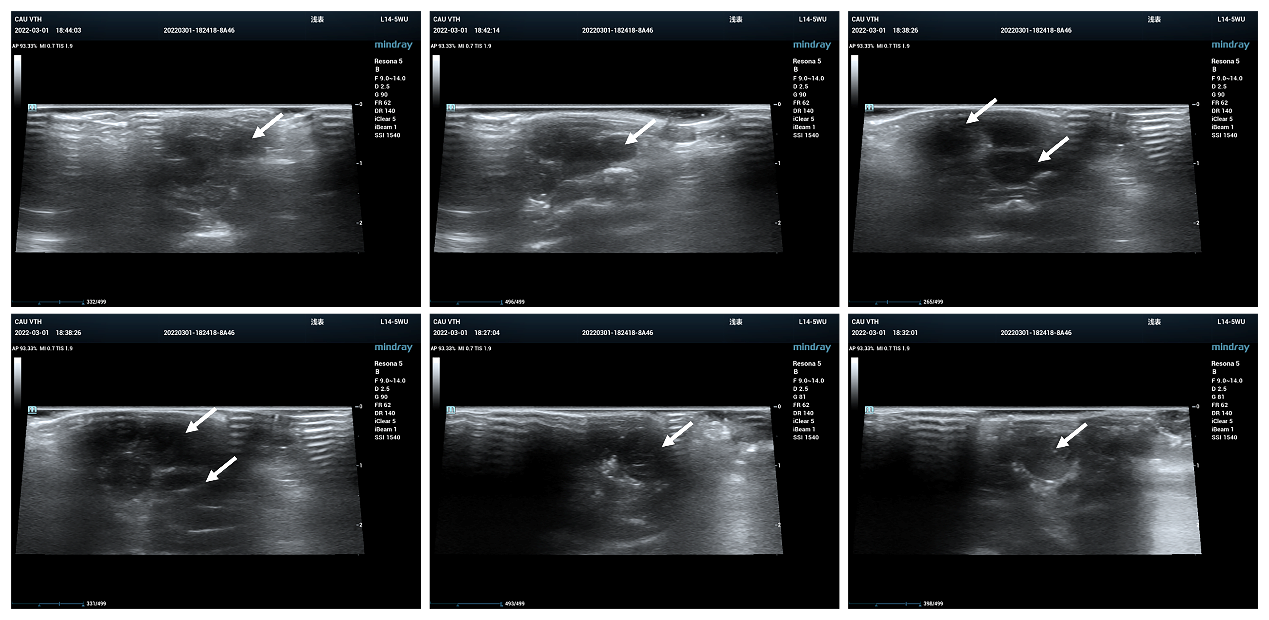


**Supplementary Figure 1. Pregnancy identification of ICR mice by ultrasound.** Multiple female ICR mice were caged with one male ICR mouse and the female mice with vaginal plugs were selected the next day. The pregnancy status of the mice was confirmed by ultrasound 10 days later. Round and full hypoechoic gestational sac (white arrow) could be seen in the sonogram and obvious fetal heart pulsation in some gestational sac.

**Supplementary Figure 2**

**
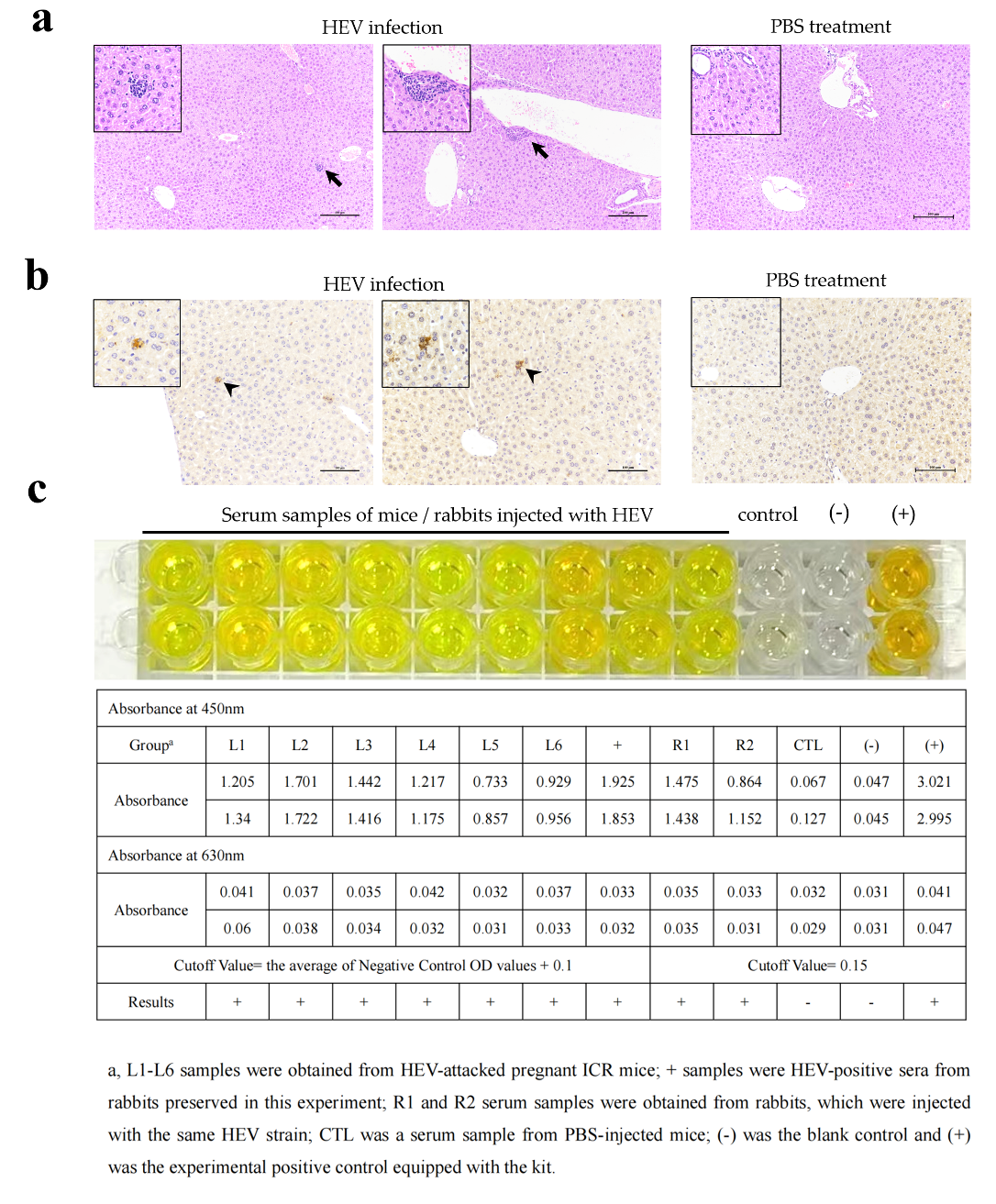
**

**Supplementary Figure 2. Detection of HEV in the liver and serum of pregnant ICR mice with HEV infection.** (a) Hematoxylin eosin-stained and (b) IHC sections of mice livers with HEV infection or PBS treatment. Inflammatory foci (arrow) dominated by lymphocytes and plasma cells could been seen in the livers of pregnant ICR mice. Positive signals for HEV ORF2 protein could been seen in IHC sections. (c) ELISA detection of serum samples from mice with HEV infection (HEV-Ab-IgG, Mouse Hepatitis E Virus IgG antibody ELISA Kit, SAB). Serum samples from mice with HEV infection were significantly positive. The last two columns were negative and positive controls for ELISA experiments.

**Supplementary Figure 3**


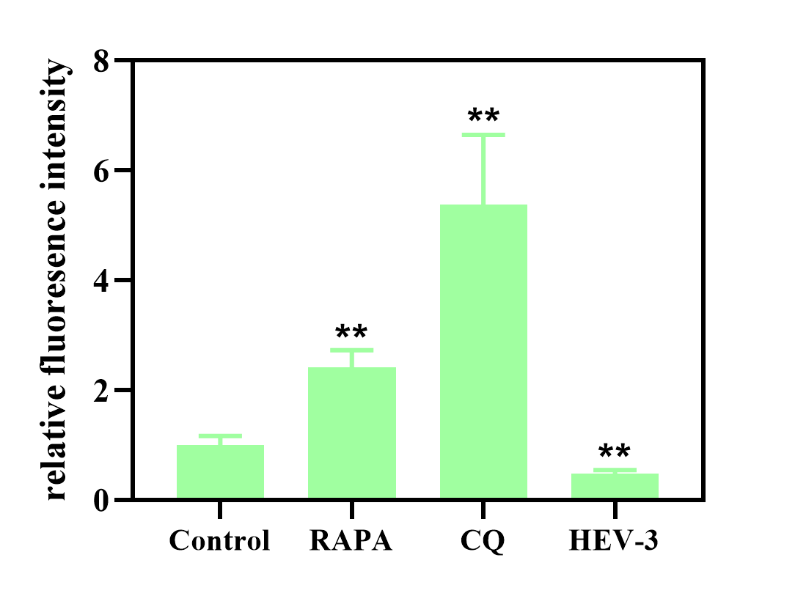


**Supplementary Figure 3. Quantitative analysis of GFP fluorescence signals in JEG-3 cells.** The fluorescence signal of phagosome-GFP in JEG-3 cells after 24 h treatment with PBS, RAPA (400 nM), CQ (50 μM) and HEV (1×10^4^ copies). Bars indicate mean ± SEM, *n* = 3. ^*^*P* < 0.05

**Supplementary Figure 4**


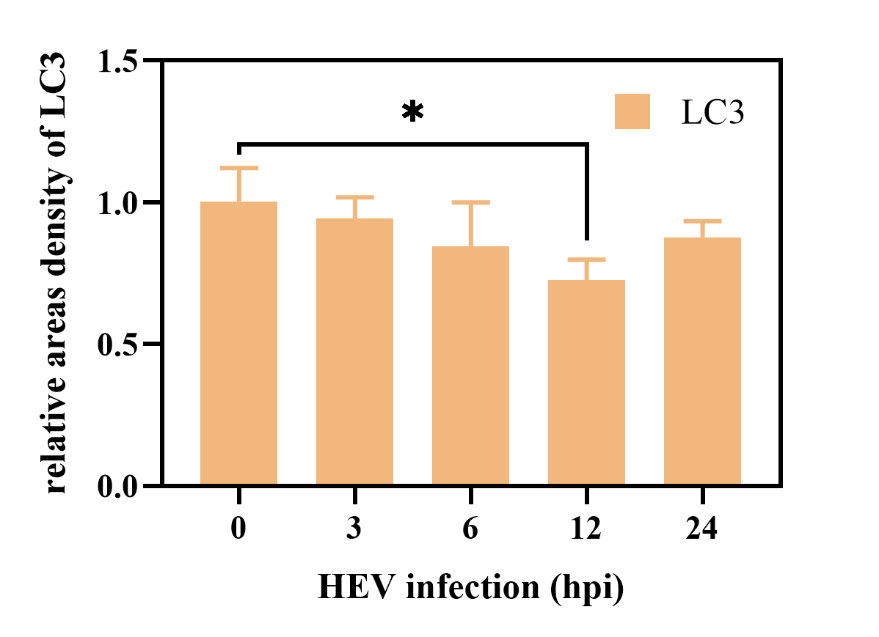


**Supplementary Figure 4. Quantitative analysis of LC3 IHC in mouse placenta.** The expression of LC3 protein in placenta of mice with 12 hpi HEV infection was significantly lower than that of the control group. Bars indicate mean ± SEM, *n* = 3. ^*^*P* < 0.05

**Supplementary Figure 5**


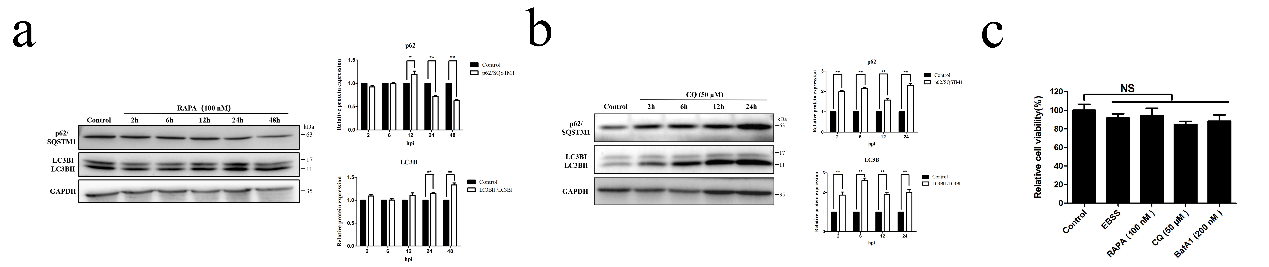


**Supplementary Figure 5. Effective JEG-3 cells autophagy induction and inhibition with drugs treatment.** Western blot of LC3 and p62 in JEG-3 cells with different time (a) RAPA (100 nM) and (b) CQ (50 μM) treatment. (c) MTT results of JEG-3 cells treated with specific concentrations of RAPA and CQ for 24 h. RAPA and CQ treatment at the indicated concentrations for 24 h can successfully construct models of induction or inhibition of autophagy, and has no significant effect on the proliferation of JEG-3 cells. Bars indicate mean ± SEM, n = 3. ^*^*P* < 0.05, ^**^*P* < 0.01

**Supplementary Figure 6**


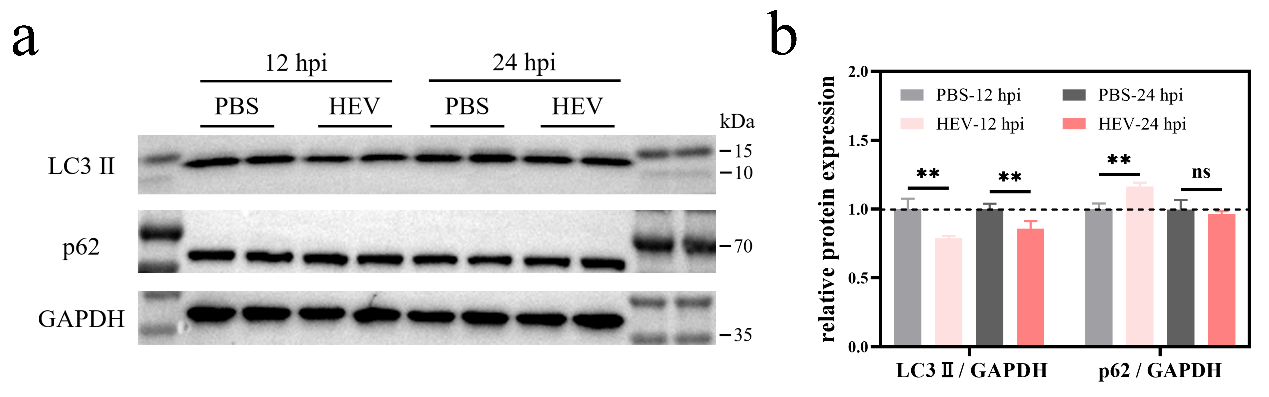


**Supplementary Figure 6. Inhibition of autophagy in JEG-3 cells with HEV inoculation.** (a) Western blot graphs and (b) quantitative analysis of LC3 and p62 in JEG-3 cells with HEV inoculation after 12 h and 24 h. Acute infection with HEV inoculation (12, 24 hpi) resulted in significant inhibition of autophagy in JEG-3 cells. This part of the data was provided to complement the expression of LC3 and p62 proteins in the control group at a specific time. Bars indicate mean ± SEM, n = 3. ^*^*P* < 0.05, ^**^*P* < 0.01

**Supplementary Figure 7**


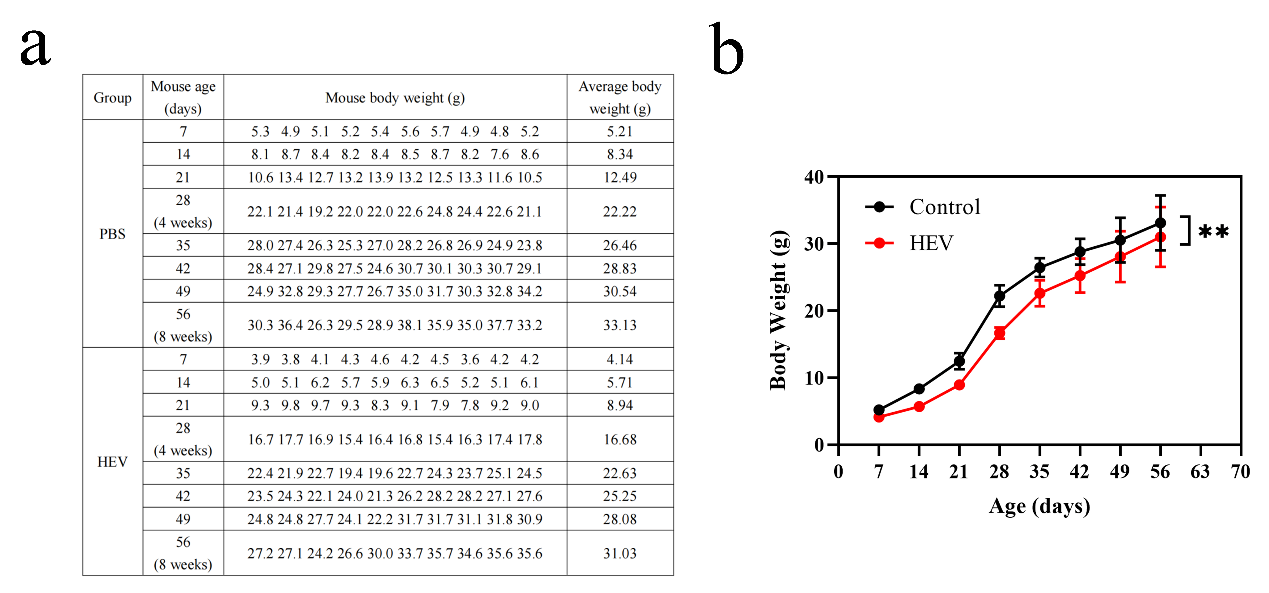


**Supplementary Figure 7. Mother mice with HEV injection produced a significantly slower body weight gain in their litters.** (a) The table and (b) the graph of body weights recorded during two months in control and experimental newborn mice. The weight of newborn mice in the experimental groups were significantly lower than that of the control groups. n = 10. ^*^*P* < 0.05, ^**^*P* < 0.01
